# Effect of health education on food hygiene behavior among food providers in India

**DOI:** 10.6026/973206300222063

**Published:** 2026-04-30

**Authors:** Durgesh Kumar Sharma, Rashmi Pilkhwal, Shweta Shrivastava

**Affiliations:** 1 Department of Community Medicine, Gandhi Medical College, Bhopal, Madhya Pradesh, India; 2 Department of Community Medicine, Atal Bihari Vajpayee Government Medical College, Vidisha, Madhya Pradesh, India

**Keywords:** Food safety (FS), public health, food handlers (FHS), food hygiene behavior, food-borne diseases

## Abstract

Poor food hygiene practices among food handlers contribute significantly to food-borne diseases and public health burden. Therefore, it is of interest to assess the food hygiene behavior of food suppliers working within the campus of Gandhi Medical College and Hamidia Hospital to determine the difference between pre- and post-test scores. This was a quasi-experimental pre- and post-educational intervention study conducted on the campus of Gandhi Medical College and Hamidia Hospital in Bhopal over 3 months. Descriptive statistics were depicted and McNemar's test was calculated on pre- and post-test proportions. The educational intervention significantly improved food handlers'understanding of food hygiene behavior, which is crucial for minimizing the risk of foodborne illnesses. Thus, we show that the food handlers exhibited inadequate food hygiene practices during the study.

## Background:

Food Safety (FS) is the assurance that food will not cause any harm to the consumer when it is prepared or consumed according to its intended use. Food safety is one of the most important factors in public health and contributes significantly to health care costs [1, 2]. On 7 April 2015, WHO adopted the World Health Day theme "From farm to plate, make food safe," highlighting the difficulties in ensuring food safety at all stages [3, 4]. A variety of stakeholders, including food handlers, food business operators (FBOs), customers, legislators and the government, play a part in ensuring that people are protected from the dangers of consuming contaminated food. In this regard, the Food Safety and Standards Act (FSSA, 2006) created the Food Safety and Standards Authority of India (FSSAI) in 2008 as an agency of the Ministry of Health and Family Welfare, Government of India [5, 6- 7]. Food handlers (FHS) may contaminate food by actions associated with ignorance of the fundamentals of FS, such as cross-contamination; appropriate cooking and storage temperatures and personal hygiene [8, 9-10]. Improper food handling by food providers during food production processes, such as cooking, storing and serving, can play a major role in food contamination, leading to food-borne diseases [11, 12, 13- 14]. Therefore, it was of interest to assess the food hygiene behavior of food suppliers in the Gandhi Medical College and Hamidia Hospital campus and to determine the difference between pre- and post-test assessments.

## Methodology:

## Study design and setting:

A quasi-experimental pre- and post-educational intervention study was conducted in Gandhi Medical College and Hamidia Hospital campus in Bhopal, which has several food handlers serving students, employees, patients and attendants.

## Study duration:

3 months

## Study population:

It consisted of food handlers who were involved in the preparation, handling, or serving of food in food vending establishments located within the campus of Gandhi Medical College and Hamidia Hospital.

## Inclusion criteria:

Food handlers who gave consent to participate in the study

## Sample size and sampling technique:

A census method was adopted. All food handlers working on campus during the study period were approached to participate. Out of these, 46 food handlers consented and were included in the final analysis. Among these 46 food handlers, 18 were owners of the food establishments.

## Study tool:

Three sections of a structured, closed-ended questionnaire were used to gather data:

Socio-demographic details (age, gender, education, marital status, socio-economic status, and years of work experience). Food hygiene and personal hygiene practices (handwashing, fingernail cleanliness, use of apron, chewing gum while handling food, coughing/sneezing practices, avoidance of food handling while ill). Environmental hygiene practices (cleanliness of food service area, health check-up status, food safety training, license status). Each behavior-related item was assessed as a binary response (Yes/No).

## Development and validation of the questionnaire:

The questionnaire was developed after an extensive review of existing literature and previously published studies on food hygiene practices. Content validity was ensured by review from subject experts in Community Medicine. The questionnaire was pre-tested among a small group of food suppliers outside the study area to assess clarity, relevance and feasibility. Necessary modifications were made in response to feedback before the final administration. The study flow is depicted in Figure 1 (see PDF).

## Educational intervention:

The food providers received one-to-one educational intervention following the pre-test assessment. The intervention included detailed discussions on the significance of food-borne illnesses and food safety, proper hand-washing, nail care, wearing clean clothes and wearing aprons, safe food handling techniques (not chewing gum, not coughing or sneezing when preparing food, not handling food when sick), cleanliness and environmental hygiene in spaces used for food preparation and serving and the significance of routine health examinations and instructions in food safety. The investigators led the sessions. The duration of the session was around twenty to thirty minutes.

## Post-test assessment:

A post-test assessment using the same questionnaire was conducted after one month of the educational intervention to assess changes in food hygiene behavior.

## Data collection:

It was done in digital mode (Google Form) under the guidance of mentors before (Pre-Test) and after (Post-Test) the educational program (one-to-one interaction).

Ethical clearance was obtained from the Institutional Ethics Committee, Gandhi Medical College, Bhopal (Letter No. 16955/MC/IEC/2025, dated 29/04/2025).

## Statistical analysis:

The results were expressed in percentages, percentage change and McNemar's test was calculated on pre- and post-test scores.

## Results:

The study's results illustrate the distribution of 46 food handlers by various socio-demographic characteristics. The majority were male (38; 82.6%) and most participants belonged to the 41-50 years age group (16; 34.8%). The mean age of the food handlers was 45.2 ± 9.9 years. Regarding educational status, 15 (32.6%) had completed primary school. Most of the food handlers were married (37; 80.4%). As per the Modified Kuppuswamy socio-economic classification, the majority belonged to the Upper Lower class (28; 60.8%) (Table 1 - see PDF). Most of the food suppliers 17 (36.9%) had >15 years of working experience (Figure 2 - see PDF). Food safety training was not received by 38 (82.7%) of food handlers and those 8 (17.3%) had received the training more than 10 years ago (Figure 3 - see PDF).Health check-up in the last six months was not done by 30 (65.2%) of the food handlers (Figure 4 - see PDF). License status was assessed at the level of food vending facilities rather than at the level of individual food handlers, since a single vendor engaged several food suppliers. So out of 18 vendors, 11 (61.1%) of them did not have the licence to practice as a vendor (Figure 5 - see PDF).

The difference between pre- and post-test assessments was assessed using McNemar's test. The percentage of food handlers who wore clean clothes improved from 13 (28.2%) before the educational intervention to 20 (43.4%) following it and this change was statistically significant (p=0.008). There was a statistically significant improvement in fingernail cleanliness, from 12 (26%) to 30 (65.2%) (p<0.05) and hand washing practices also improved significantly, from 20 (43.4%) in the pre-test to 27 (64.7%) in the post-test (p=0.008). The behaviour of chewing gum while preparing or serving food was statistically significantly reduced in terms of food handling practices, going from 15 (32.6%) to only 5 (10.9%) food handlers chewing gum, following the intervention (p<0.05). The number of food handlers who coughed or sneezed while cooking or serving food decreased from 13 (28.2%) to 8 (17.6%) and this result was statistically significant (p=0.02). Food suppliers who wore aprons while cooking improved slightly, from 11 (23.9%) to 14 (30.4%) and those who avoided serving food when ill increased from 11 (23.9%) to 14 (28.3%), but none of the improvements were statistically significant (p>0.05). In terms of environmental conditions, the cleanliness of the food service area among food suppliers increased from 27 (58.8%) before the educational intervention to 32 (69.5%) following it and this difference was statistically significant (p=0.02), as depicted in Table 2 (see PDF).

## Discussion:

The effectiveness of a health education package on hand hygiene knowledge, attitude and practices among food handlers in a tertiary care hospital in Delhi were assessed by Chauhan *et al.* [15] in 2021-22. The maximum improvement, from 6.3% to 81.1%, was observed in handwashing for a duration of >20 seconds (P < 0.05). This improvement, in particular, was observed in domains such as fingernail cleanliness and handwashing practices in the present study. In India, behavioural factors and determinants have also been studied. Even after adjusting for education and experience, a 2025 study conducted at a tertiary care hospital in New Delhi by Chauhan *et al.* [16] found that training was the strongest predictor of higher KAP scores, despite food handlers having weak baseline knowledge, attitudes and practices. This supports the findings of the current study, in which those who had previously undergone food safety training were uncommon, highlighting training as a critical lever for improvement. Crucially, following the intervention, the unwanted habit of chewing gum while cooking or serving food decreased dramatically (from 32.6% to 10.9%), highlighting the value of health education in changing improper behaviour. Risky behaviours like coughing or sneezing while handling food also decreased (from 28.2% to 17.6%). Similarly, avoiding food handling when unwell (23.9% to 28.3%) and using an apron (23.9% to 30.4%) did not have statistical significance in pre- and post-test scores; the rising trends indicate beneficial behavioural adjustments. This aligns with a study by Sobhan *et al.*[17] (2022) indicating that educational initiatives can effectively change behaviors and attitudes towards hygiene in food service settings. In a study by Surwase *et al.*[18], when assessing hygiene practices, 74% of handlers had clean nails and approximately 50% washed their hands with soap and water after using the toilet.

The cleanliness of kitchen areas was maintained in 73% of establishments, with 62% using soap and detergents for cleaning. A 2024 cross- sectional assessment by Singh and Singh [13] in Kanpur reported that although food handlers demonstrated satisfactory knowledge and attitudes towards hygiene, actual practices were notably poor, with only a small proportion consistently engaging in key hygienic behaviours, such as hand washing before food handling. The trend observed in this study, where baseline habits were unsatisfactory despite apparent awareness, is noteworthy because awareness did not always translate into safe practice. Evidence from Sangli, Maharashtra, where a 2025 study by Dhudum and Bhosale [12] found that even when a moderate proportion of street food sellers had intermediate food hygiene knowledge, there was a weak association with actual sanitary practices, highlighting the gap between knowledge and practice among food handlers. This is consistent with the findings of this study, in which post-intervention improvement in structural behaviours (such as donning aprons or refraining from touching food while unwell) was relatively low. The study is limited to food vendors providing food on a single medical college and hospital campus, which l imits the generalizability of the findings to other contexts, such as community-based food establishments or commercial food outlets. The study did not involve microbiological testing of food samples, which could have objectively validated the stated practices. The results of this study show that while short-term educational interventions can provide measurable improvements in specific hygiene behaviours, institutional reinforcement through organised systems is necessary for sustainable compliance. SOPs for food handling should be created and implemented. Comprehensive training for food handlers focusing on essential food hygiene practices should be implemented. Routine medical examinations should be made mandatory, as per WHO and FSSAI guidelines, to prevent the spread of diseases through food. Authorities should ensure the availability of protective clothing (aprons, head covers, gloves) at affordable prices and enforce their use through inspections. Fumigation of the vending sites at regular intervals and alternate arrangements to provide food during that time should be ensured. Feedback from both staff and consumers regarding food safety practices should be collected. Health education campaigns that target both vendors and consumers can increase demand for hygienic practices, thereby encouraging food suppliers to maintain higher standards.

## Conclusion:

We show a marked increase in food suppliers' understanding of food hygiene behavior following an educational intervention, which is crucial for minimizing the risk of foodborne illnesses. However, while the study confirms the positive impact of health education, it also underscores the necessity for resources to bring improvements. Behavioral change in food hygiene is not solely dependent on education; it requires a multifaceted approach that includes adequate facilities, consistent monitoring and strict enforcement of SOPs.
